# Increased Expression of Prolyl Endopeptidase Induced by Oxidative Stress in Nucleus Pulposus Cells Aggravates Intervertebral Disc Degeneration

**DOI:** 10.1155/2022/9731800

**Published:** 2022-04-13

**Authors:** Huo-Liang Zheng, Wen-Ning Xu, Peng-Bo Chen, Lei-Sheng Jiang, Xin-Feng Zheng, Sheng-Dan Jiang

**Affiliations:** ^1^Department of Clinic of Spine Center, Xinhua Hospital, Shanghai Jiaotong University School of Medicine, Shanghai 200082, China; ^2^Department of Spinal Surgery, Orthopedic Medical Center, Zhujiang Hospital, 6 Southern Medical University, Guangzhou 510280, China

## Abstract

A healthy microenvironment of the intervertebral disc tissue is characterized by hypoxia owing to its sparse vascular distribution. Oxidative stress plays a pivotal role in the pathological development of intervertebral disc degeneration (IVDD). We found that the expression of prolyl endopeptidase (PREP) increased in degenerative nucleus pulposus (NP) tissues. The purpose of this study was to determine whether PREP is involved in oxidative-stress-induced IVDD. Tertbutyl hydroperoxide can inhibit the expression of PREP by activating the PI3K/AKT signaling pathway at low concentrations in NP cells. Knockdown of PREP protected NP cells from apoptosis induced by oxidative stress, whereas overexpression of PREP exacerbated the apoptosis of NP cells. We also investigated the connection between the PI3K/AKT signaling pathway and PREP and found that the activation of the PI3K/AKT signaling pathway downregulated the expression of PREP by inhibiting p53. As a crucial transcription factor, p53 binds to the PREP promoter region and promotes its transcription. Overexpression of PREP also impairs protein secretion in the extracellular matrix of NP cells. Furthermore, the in vivo knockout of PREP could attenuate puncture-induced IVDD. These findings suggested that the downregulation of PREP might maintain the viability of NP cells and attenuate IVDD under oxidative stress.

## 1. Introduction

Intervertebral disc degeneration (IVDD) is the leading cause of low back pain (LBP), which not only limits the patient's activities but also creates a huge social and economic burden [1–4]. A healthy intervertebral disc is mainly composed of the central nucleus pulposus (NP) and annulus fibrosus, which is wrapped around the NP. NP cells originate from the embryonic notochord and play an irreplaceable role in maintaining intervertebral disc homeostasis [5, 6]. Studies have shown that NP cells are similar to chondrocytes in terms of morphology and function. Type II collagen and proteoglycans secreted by NP cells maintain the elasticity of intervertebral discs [7, 8].

IVDD often occurs initially in NP cells. Owing to the altered normal physiological processes of NP cells, the components of the extracellular matrix (ECM) also change accordingly [9]. During IVDD, NP cells are gradually replaced by fibrocartilage-like cells. This also results in reduced secretion of the normal ECM, which contains proteoglycans and type II collagen; however, the secretion of the fibrotic matrix increases pathologically. Eventually, the structure of the intervertebral disc changes and loses its original elasticity. There are many causes of disc degeneration, including trauma, strain, inflammation, stress, age, and genetics [10–12].

Recent research has found that oxidative stress is a critical factor in IVDD [13]. The microenvironment of healthy intervertebral disc tissue is hypoxic, and the generation and scavenging of intracellular reactive oxide species (ROS) are dynamically balanced under physiological conditions [14]. Oxidative stress occurs when this equilibrium is disturbed [15, 16] and causes increased intervertebral disc apoptosis and senescence, as well as decreased autophagy; it also induces damage to the matrix structure and promotes degradation of the intervertebral disc tissue matrix [17–19]. As a result, the elasticity of the intervertebral disc is significantly reduced, while the stiffness is significantly increased, impairing the mechanical function of the intervertebral disc and thereby triggering disc degeneration.

Prolyl endopeptidase (PREP), also known as prolyl oligopeptidase, is a member of serine proteases [20]. PREP can hydrolyze the C-terminus of proline residues of prolyl-containing peptides smaller than 3 kDa [21]. Furthermore, PREP plays an essential role in many physiological processes, such as the regulation of peptides and hormones. Emerging evidence suggests that PREP is closely related to diseases such as Alzheimer's disease, schizophrenia, and diabetes [22, 23]. N-Ac-PGP generated from PREP promotes the inflammatory response in degenerative discs, indicating a potential association between PREP and IVDD [24, 25]. However, the function of PREP in IVDD has not been investigated.

In this study, PREP was upregulated in degenerated disc tissues, which was verified by sequencing analysis. The expression of PREP was also significantly reduced by stimulation with tert-butyl hydroperoxide (TBHP) at low concentrations. Further studies proved that this reduction was caused by the activation of the PI3K/AKT signaling pathway in NP cells. Moreover, PREP was found to be related to the apoptosis of NPs induced by TBHP. In degenerated intervertebral disc tissues, the expression of PREP was consistent with that of p53. We found that p53, which is also a transcription factor downstream of the PI3K/AKT pathway, modulated the transcription of PREP in NP cells. In a mouse model of puncture-induced disc degeneration, the PREP-knockout (PREP-KO) group showed slight disc degeneration compared to that in the wild-type (WT) group. This study demonstrates that PREP is an unrecognized target for the treatment of IVDD.

## 2. Materials and Methods

### 2.1. Clinical Tissue Collection

The Ethics Committee of Xinhua Hospital, affiliated with the Shanghai Jiaotong University School of Medicine, reviewed and approved this study. All patients read and signed an informed consent form before surgery. Degenerative disc tissues were collected from patients undergoing lumbar spine surgery for lumbar disc herniation and spinal stenosis (13 males and 12 females, 27–69 years old). Normal disc tissues were collected from patients with congenital hemivertebrae deformity (one female and two males, 14–22 years old). All patients underwent magnetic resonance imaging, and the grade of disc degeneration was determined based on the Pfirrmann classification system (I–V).

### 2.2. NP Cell Isolation and Culture

After collecting the NP specimens during surgery, we transferred the tissue to a clean bench using a sterile centrifuge tube and rinsed it three times with phosphate-buffered saline (PBS, Cytiva). Next, the NP tissue was digested with type II collagenase (0.2%, Sigma-Aldrich) for 4 h at 37°C. After centrifugation, the supernatant was removed, and the tissue was resuspended and cultured in DMEM/F12 medium (Corning) with 10% fetal bovine serum (FBS, SANTACRUZ). Adherent NP cells were observed under a microscope after five days of culture without replacing the medium. When the cell density reached 80–90%, we passaged the NP cells using Trypsin-EDTA (Biosharp), and the passaged NP cells were the first passage. Only NP cells within four passages were used for in vitro studies.

### 2.3. In Vitro Oxidative Stress Model

Excess of TBHP can cause an increase in ROS in NP cells, triggering oxidative stress [18]. The in vitro oxidative stress model was established by treating NP cells with 400 *μ*M TBHP for 6 h when the cell density reached 70–80%. The cells were collected for further studies.

### 2.4. Immunohistochemistry Staining

NP tissues were fixed with 4% paraformaldehyde and embedded in paraffin. After the embedded tissues were cut into sections, xylene and alcohol were deparaffinized and rehydrated. Endogenous peroxidase activity was blocked with methanol-diluted hydrogen peroxide. After antigen retrieval with pepsin (MKBio), 5% bovine serum albumin (BSA) was used to block nonspecific binding sites on the sections. Next, the tissues were incubated with the primary antibody overnight at 4°C. PBS was used to remove unbound primary antibodies, and the target antigen was detected using an HRP-DAB kit (Maxvision™2). Images were obtained using an Olympus microscope.

### 2.5. Immunofluorescence Staining

The cells were washed with PBS, which was followed by formaldehyde fixation. The cell membranes were then ruptured using Triton X-100 (Beyotime) and blocked with 5% BSA for 1 h. The NP cells were incubated with the primary antibody overnight at 4°C. TBST was used to remove the unbound primary antibodies. A secondary antibody with a fluorescent label (Beyotime) was added to detect the target antigens bound by the primary antibodies. DAPI (Beyotime) and FITC-conjugated phalloidin (MKBio) were used to stain the nuclei and cytoskeletons. Images were obtained using an Olympus microscope.

### 2.6. Western Blotting

NP cells were lysed with RIPA buffer (Genecome) supplemented with phenylmethanesulfonyl fluoride (SANTACRUZ) at 4°C after washing with PBS. After centrifugation and aspiration of the supernatant, the protein and loading buffer were mixed and boiled for 10 min. After electrophoretic separation, the proteins on the SDS-PAGE polyacrylamide gels were transferred to PVDF membranes (Beyotime). After blocking with nonfat milk (5%) for 1 h at room temperature, the membranes were incubated with primary antibodies against PREP (Proteintech, 11536-1-AP), Bcl-2 (Cell Signaling Technology, 3498), Bax (Cell Signaling Technology, 14796), P53 (Cell Signaling Technology, 2527), MMP-3 (Proteintech, 17873-1-AP), and aggrecan (Abcam, ab3778) overnight at 4°C. After washing three times with TBST to remove the unbound primary antibodies, the membranes were incubated with horseradish peroxidase-conjugated secondary antibodies (Epizyme). Finally, antibody–antigen complexes were detected using an ECL reagent (Epizyme).

### 2.7. Quantitative RT-PCR

After lysis with TRIzol (Beyotime), the lysates were mixed with chloroform. Next, the samples were centrifuged, and the upper RNA-containing transparent liquid was extracted. After mixing with an equal volume of isopropanol, the samples were centrifuged to obtain RNA pellets. RNA was washed with 75% ethanol and reverse-transcribed into cDNA using PrimeScript™ RT Master Mix (TAKARA) after centrifugation. Real-time polymerase chain reaction (RT-PCR) was carried out using PCR Master Mix (Yeasen). GAPDH was used to normalize the expression of target genes. Primers used for PCR are shown in Table 1.

### 2.8. Chromatin Immunoprecipitation (ChIP) Assay

First, 1% formaldehyde (Beyotime) was used to crosslink the NP cells, which was stopped by glycine. NP cells were collected and resuspended in lysis buffer (Beyotime) on ice after washing with PBS. Next, the lysates were sonicated to obtain DNA fragments. The debris was pelleted and removed by centrifugation, and the supernatant was collected. Chromatin samples were diluted, and protein A + G Agarose/Salmon Sperm DNA was used to reduce nonspecific binding. After centrifugation, the supernatant was collected and part of it was stored as input. Then, the primary antibody was incubated with the supernatant at 4°C overnight. Protein A + G Agarose was added to the supernatant, the mixture was centrifuged, and the supernatant was removed. Elution buffer was added to the precipitate, and the supernatant was collected. After decrosslinking the DNA-protein complex with NaCl, we added ribonuclease A. Proteinase K, ethylenediaminetetraacetic acid, and Tris-HCl were added and incubated with samples at 45°C for 1 h. The purified DNA was then prepared for the study. Primer sequences for ChIP are shown as follows:

ChIP-F: CACGAGGTGGGAACTGGAAT

ChIP-R: GCCTCACCCTTAGTTCACCA

### 2.9. In Vitro Transfection of Lentivirus or siRNA

Cells were seeded at a density of 30% in a six-well plate before the lentivirus transfection. Lentivirus encoding PREP or P53 was added to serum-free DMEM/F12 medium of NP cells in the presence of polybrene. One day after transduction, the culture medium was replaced with a normal DMEM/F12 medium supplemented with 10% FBS. Cells were selected using puromycin (Beyotime) 48 h after transfection. Overexpression lentivirus was constructed using pGMLV-CMV vector plasmid. For the siRNA transfection, cells were plated in a six-well plate at 30–50% density. Lipofectamine 2000 (Thermo Fisher Scientific) and siRNA were diluted in serum-free DMEM/F12 medium. Five minutes later, siRNA and Lipofectamine 2000 diluent were mixed and allowed to stand at room temperature for 20 min. The mixture was then added to NP cells for transfection. The culture medium was replaced with a normal DMEM/F12 medium with 10% FBS one day later. The siRNA sequences are shown as follows:

siRNA1: CCAUGCUUGGACCACUGAUUA

siRNA2: CGCUAUGUCUUGUUAUCAAUA

siRNA3: CCCAACAUACUGUCUGACGAU

### 2.10. Flow Cytometry Analysis

The NP cells were collected after digestion with 0.25% trypsin. After washing with PBS, the cells were pelleted via centrifugation and resuspended in 100 *μ*l binding buffer. Then, 5 *μ*l annexin V-FITC (Beyotime) and 10 *μ*l propidium iodide (PI, Beyotime) were added and incubated with the cells for 20 min in the dark. The cells were immediately analyzed via flow cytometry (Beckman CytoFLEX).

### 2.11. Dual-Luciferase Reporter Assay

P53 binding sequence was predicted in 803 bp to 809 bp upstream of the PREP promoter regions. The binding sequence in PREP promoter regions was mutated from TATGCCC to GCGTAAA. Wild-type or mutant PREP promotor plasmids were constructed with the pGL3-Basic vector. Renilla-containing pRL-TK plasmid was used to normalize transfection efficiency. Lipofectamine 3000 (Thermo Fisher Scientific) was used for the transfection. The NP cells were collected and plated in 96-well plates 24 h after transfection. Luciferase activity in NP cells was detected using a dual-luciferase reporter assay kit (Promega) 24 h after treatment.

### 2.12. Animals

The Ethics Committee of the Xinhua Hospital reviewed and approved the animal experiments. Female WT C57BL/6J and PREP-KO mice (aged 12 weeks, 20–25g) were obtained from Shanghai Model Organisms Center, Inc. The gene knockout mouse was constructed with CRISPR/Cas9 technology. The shifting of open reading frame in PREP causes its disfunctiondysfunction. The PREP KO mice used in this study carry a partial deletion of exon 3 of the PREP gene. All mice were housed in pathogen-free facilities under a 12 -h light and 12 -h dark cycle. Twelve mice were randomly divided into two groups of six, and six PREP-KO mice were grouped individually. The mice were anesthetized with 1% pentobarbital sodium (0.1mg/kg). The mice in the control group were only punctured with a needle on the skin of the tail, without damaging the intervertebral disc. The mice in the WT or PREP-KO groups were punctured with a 23G syringe needle at the coccygeal 4-5 disc level. The needle was inserted into the center of the NP, rotated 360°, and held for 30 s. The mice were euthanized six weeks after surgery, and the tissues were collected for further use. The primer sequences for identifying WT and PREP-KO mice are as follows: Primer1: TACCGCTACCCCTGCTTCA; Primer2: GCTATGTCGGCTCCAACCA; Primer3: AGCTACTTCCTGCCCCCTCTTAC; Primer4: GGAATCCCCAACACTGACACAAA.

### 2.13. Bioinformatics Analysis

GSE146904 and GSE167931 were obtained from the GEO database. The limma package was used for data analysis.

### 2.14. Statistical Analysis

Data are presented as mean ± SD. Differences were analyzed by a two-tailed Student's *t*-test for comparisons between two groups, and one-way analysis of variance was used for comparisons between multiple groups. Least significance difference was used for post hoc test. Differences were considered statistically significant at *P* < 0.05.

## 3. Results

### 3.1. Upregulation of PREP in Degenerated Intervertebral Disc Tissues

**Figure 1:**
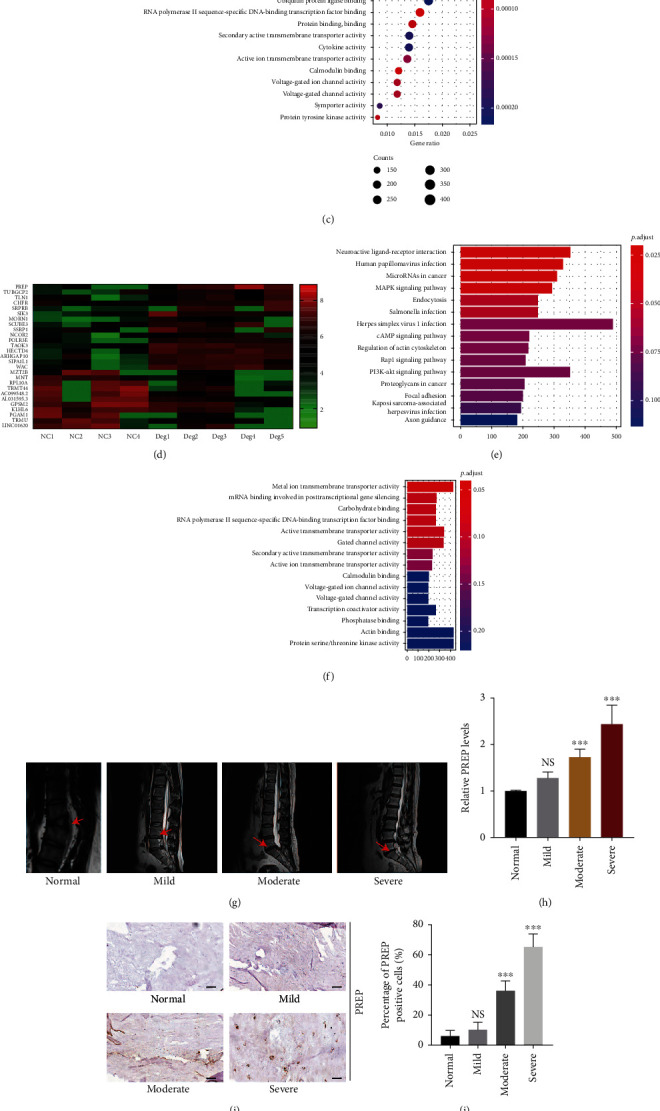
PREP is upregulated in degenerated intervertebral disc tissues. (a) Heat map of the associated genes in GSE146904. Red represents upregulation. Green represents downregulation. (b) KEGG pathway analysis showed the changed function of PI3K-AKT pathway in GSE146904. (c) Go enrichment analysis of GSE146904. (d) Heat map of the associated genes in GSE167931. Red represents upregulation while green represents downregulation. (e) KEGG pathway analysis of GSE167931. (f) Go enrichment analysis of GSE167931. (g) Representative MRI of human spine graded by the Pfirrmann system. (h) The expression of PREP in human NP tissues was analyzed by qRT-PCR. Results are shown as mean ± SD, *n* = 5, ^∗∗∗^*P* < 0.001. (i) Immunohistochemical staining of PREP in human NP tissues with different degrees of degeneration. Scale bar, 50 *μ*m. (j) Quantitative of PREP positive cells. (k) Western blotting analysis of PREP in human NP tissues with different degrees of degeneration.

Ten intervertebral disc specimens were collected for sequencing, and patients with spondylolisthesis were included in the control group (GSE146904). Bioinformatics analysis showed that PREP was upregulated in patients with disc herniation and downregulated in patients with spondylolisthesis, as shown in the heat map (Figure 1(a)). KEGG pathway analysis indicated that the PI3K/AKT signaling pathway varied between the two groups, while GO enrichment analysis revealed that the serine kinase activity was altered (Figures 1(b) and 1(c)). Moreover, mRNA from five degenerated and four normal human NP cells was collected for further study (GSE167931). PREP was highly expressed in the degenerated group, which was consistent with the sequencing results of GSE146904 (Figure 1(d)). Changes in the PI3K/AKT signaling pathway and serine kinase activity were also involved in IVDD, as demonstrated by KEGG and GO enrichment analyses (Figures 1(e) and 1(f)). We performed qRT-PCR to verify the sequencing results. We collected intervertebral disc tissues with different grades of degeneration and found that the expression of PREP gradually increased as the degree of IVDD worsened (Figures 1(g) and 1(h)). Western blotting and immunohistochemistry intensity for PREP expression showed similar trends to the qRT-PCR assay (Figures 1(i)–1(k)).

### 3.2. Low-Concentration TBHP Stimulation Inhibits PREP Expression by Activating PI3K Signaling Pathway in NPs

**Figure 2:**
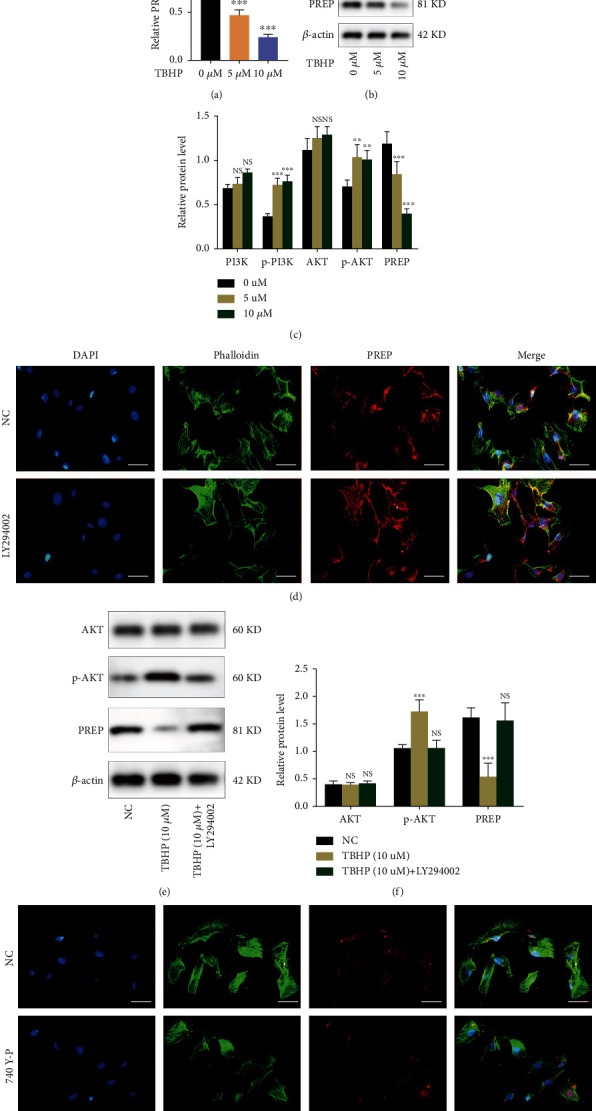
Low-concentration TBHP stimulation inhibits PREP expression by activating PI3K signaling pathway in NPs. (a) The expression of PREP in NP cells was analyzed by qRT-PCR. (b) Western blotting for the expression of PREP and PI3K/AKT signaling pathway-related proteins. (c) Quantitative analysis of the protein contents of AKT, p-AKT, PI3K, p-PI3K, and PREP. Results are shown as mean ± SD, *n* = 5, ^∗∗^*P* < 0.01, ^∗∗∗^*P* < 0.001. (d) Immunofluorescence images of PREP in human NP cells with or without LY294002 treatment. Scale bar, 50 *μ*m. (e) Western blotting for the protein levels of AKT, p-AKT, and PREP. (f) Quantitative analysis of the protein contents of AKT, p-AKT, and PREP. Results are shown as mean ± SD, *n* = 5, ^∗∗∗^*P* < 0.001. (g) Immunofluorescence images of PREP in human NP cells with or without 740 Y-P treatment. Scale bar, 50 *μ*m.

NP cells were exposed to low concentrations of TBHP for 6 h to simulate increased ROS production under normal physiological conditions. The qRT-PCR and western blotting results indicated that a low concentration of TBHP decreased the expression of PREP in NP cells in a concentration-dependent manner (Figures 2(a) and 2(b)). Moreover, the expression of p-PI3K and p-AKT was increased in NP cells exposed to TBHP, indicating the activation of the PI3K/AKT pathway (Figures 2(b) and 2(c)). Next, we used a PI3K inhibitor to investigate whether changes in PREP were related to the PI3K/AKT signaling pathway. The presence of LY294002 significantly promoted the expression of PREP in NPs, as verified by immunofluorescence analysis. Western blotting showed that LY294002 rescued the decrease in the expression of PREP induced by TBHP (Figures 2(e) and 2(f)). Activation of the PI3K/AKT signaling pathway with the PI3K agonist 740 Y-P significantly decreased the expression of PREP (Figure 2(g)). Taken together, these assays indicated that low concentrations of TBHP downregulated the expression of PREP by activating the PI3K/AKT signaling pathway.

### 3.3. PREP Promotes Apoptosis of NP Cells

**Figure 3:**
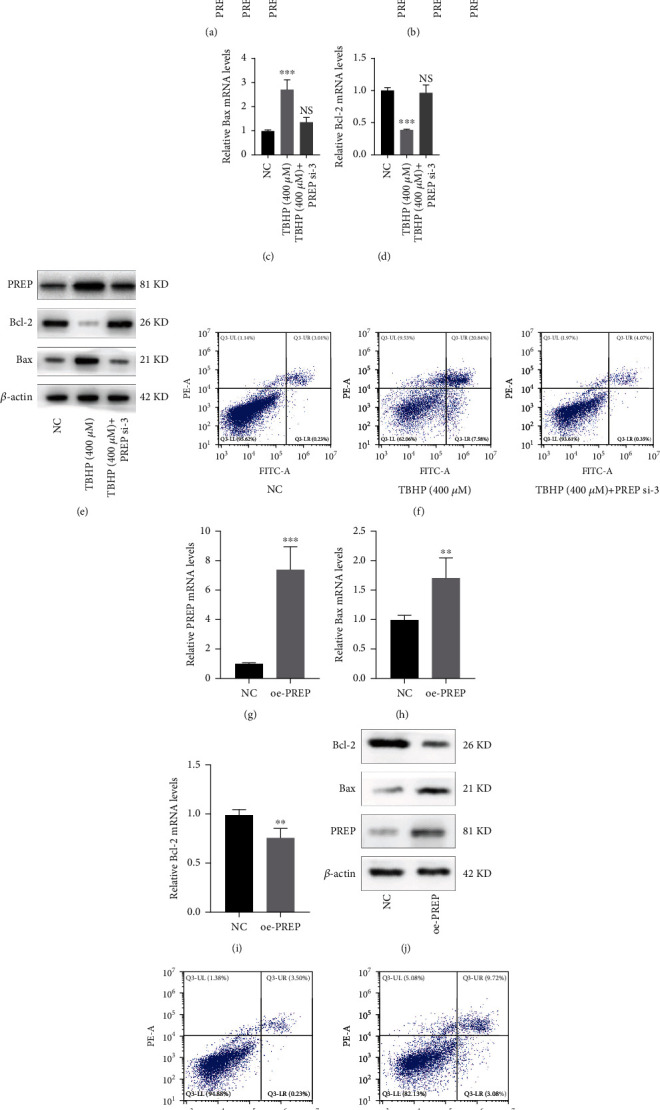
PREP promotes apoptosis of NPs. (a) The qRT-PCR measured the interference efficiency of PREP in NP cells. Results are shown as mean ± SD, *n* = 5, ^∗∗^*P* < 0.01, ^∗∗∗^*P* < 0.001. (b) Western blotting for the protein levels of PREP. (c, d) The qRT-PCR showed messenger RNA expression of Bcl-2 and Bax in NP cells. Results are shown as mean ± SD, *n* = 5, ^∗∗∗^*P* < 0.001. (e) Western blotting for the protein levels of Bcl-2 and Bax. (f) Flow cytometric analysis of apoptosis in NP cells, *n* = 3. (g–i) The qRT-PCR showed messenger RNA expression of PREP, Bcl-2, and Bax in NP cells with PREP overexpression. Results are shown as mean ± SD, *n* = 5, ^∗∗^*P* < 0.01, ^∗∗∗^*P* < 0.001. (j) Western blotting for the protein levels of PREP, Bcl-2, and Bax. (k) Flow cytometric analysis of apoptosis in NP cells with PREP overexpression, *n* = 3.

To investigate whether PREP was involved in the apoptosis of NP cells induced by oxidative stress, we constructed a small interfering RNA (siRNA) to suppress the expression of PREP, and the interference efficiency was verified via western blotting and PCR (Figures 3(a) and 3(b)). The effect of PREP silencing on apoptosis-related genes in NP cells was analyzed via PCR and western blotting (Figures 3(c) and 3(d)). It has been proved that high-concentration H_2_O_2_ inhibited PI3K/AKT pathway and caused increased apoptotic cells. We then examined whether high-concentration TBHP promotes PREP expression as the PI3K/AKT pathway was suppressed. TBHP (400 *μ*M) promoted the expression of Bax and suppressed that of BCL-2, which was attenuated in NP cells with decreased PREP expression (Figure 3(e)). Flow cytometric analysis indicated that the increase in the proportion of apoptotic cells induced by TBHP was rescued by PREP silencing (Figure 3(f)). Moreover, we constructed a lentiviral vector to overexpress PREP and studied its effect on NP cells. With the increased expression of PREP in NP cells, the expression of Bcl-2 decreased while that of Bax increased (Figures 3(g)–3(j)). Also, the overexpression of PREP significantly promoted the apoptosis of NP cells (Figure 3(k)).

### 3.4. p53 Is the Relay Baton Connecting the PI3K/AKT Signaling Pathway and PREP

**Figure 4:**
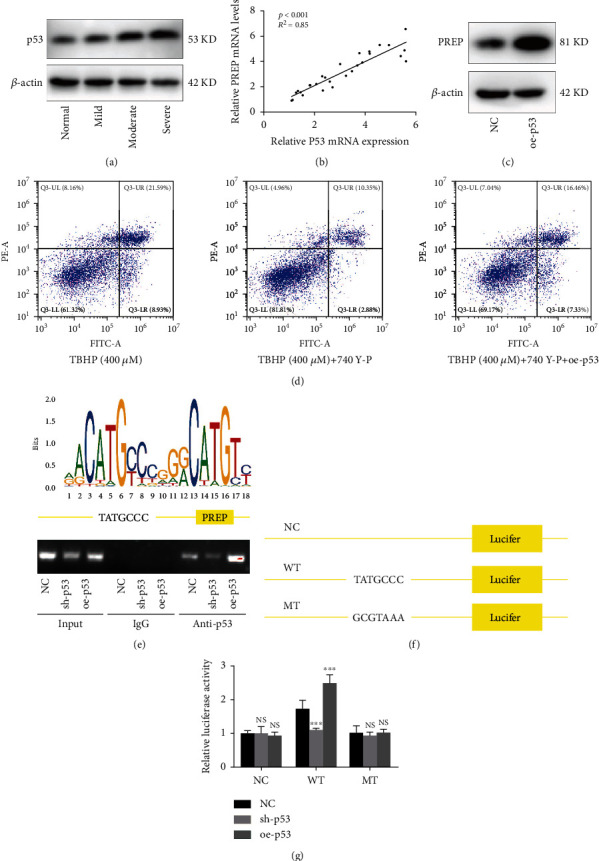
p53 is the relay baton connecting the PI3K/AKT signaling pathway and PREP. (a) Western blotting for the protein levels of p53 in human NP tissues. (b) The qRT-PCR measured the mRNA expression of p53 and PREP in human NP tissues. *n* = 28. (c) Western blotting for the protein level of PREP in NP cells with p53 overexpression. (d) Flow cytometric analysis of apoptosis in NP cells, *n* = 3. (e) Schematic illustration of p53 binding sequence and the promotor region of PREP in human. P53 could bind to sites in the PREP promotor proved by CHIP assay. (f) Mutations in the putative p53-binding site of the PREP promotor region. (g) The mutant in binding site decreased luciferase activity in NP cells. Results are shown as mean ± SD, *n* = 5, ^∗∗∗^*P* < 0.001.

As an important transcription factor downstream of the PI3K/AKT signaling pathway [26], the expression of p53 increased with the aggravation of IVDD, which was verified via western blotting (Figure 4(a)). The mRNA expression of PREP and p53 showed excellent concordance in degenerated discs (Figure 4(b)). The overexpression of p53 also promoted PREP expression, indicating that p53 may modulate the expression of PREP (Figure 4(c)). High concentration of TBHP (400 *μ*M) increased the ration of apoptotic cells. Moreover, the overexpression of p53 impaired the apoptosis of NP rescued by 740 Y-P (Figure 4(d)). We predicted p53-binding sites within the PREP promoter region in JASPAR: http://jaspar.genereg.net. Furthermore, we performed a ChIP assay to confirm that PREP transcription was regulated by p53 in NP cells. Specifically, p53 was increasingly bound to the PREP promoter site in NP cells overexpressing p53 but decreasingly bound under the suppression of p53 (Figure 4(e)). The promoter region of PREP was introduced into the luciferase reporter plasmids. We then constructed mutations in the predicted p53-binding region of the PREP promoter, and the luciferase reporter assay showed that the mutation decreased the activity of luciferase (Figure 4(f)).

### 3.5. PREP Impairs Protein Secretion in the ECM of NPs

**Figure 5:**
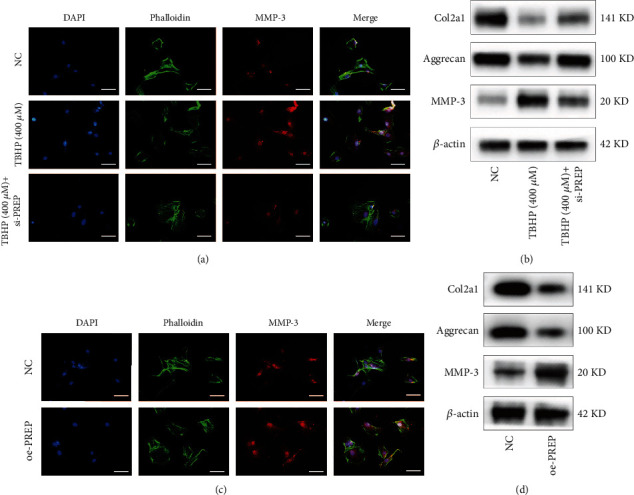
PREP impairs protein secretion in ECM of NPs. (a) Immunofluorescence images of MMP-3 in human NP cells. Scale bar, 50 *μ*m. (b) Western blotting for the protein levels of Col2a1, aggrecan, and MMP-3 in human NP cells. (c) Immunofluorescence images of MMP-3 in human NP cells with PREP overexpression. Scale bar, 50 *μ*m. (d) Western blotting for the protein levels of Col2a1, aggrecan, and MMP-3 in human NP cells with PREP overexpression.

To study the role of PREP in ECM secretion, we suppressed the expression of PREP using siRNA. TBHP (400 *μ*M) treatment significantly increased the fluorescence intensity of MMP3 in NP cells. However, this increase was greatly attenuated by the siRNA-mediated downregulation of PREP silenced by siRNA (Figure 5(a)). Western blotting indicated that TBHP suppressed the expression of Col2a1 and aggrecan while promoting the expression of MMP3, which was consistent with the immunofluorescence assay (Figure 5(b)). The expression of Col2a1 and aggrecan tended to be normal when PREP was downregulated. Furthermore, overexpression of PREP resulted in decreased expression of Col2a1 and aggrecan, which was verified by immunofluorescence and western blotting (Figures 5(c) and 5(d)).

### 3.6. PREP Knockout Protects against Puncture-Induced IVDD

**Figure 6:**
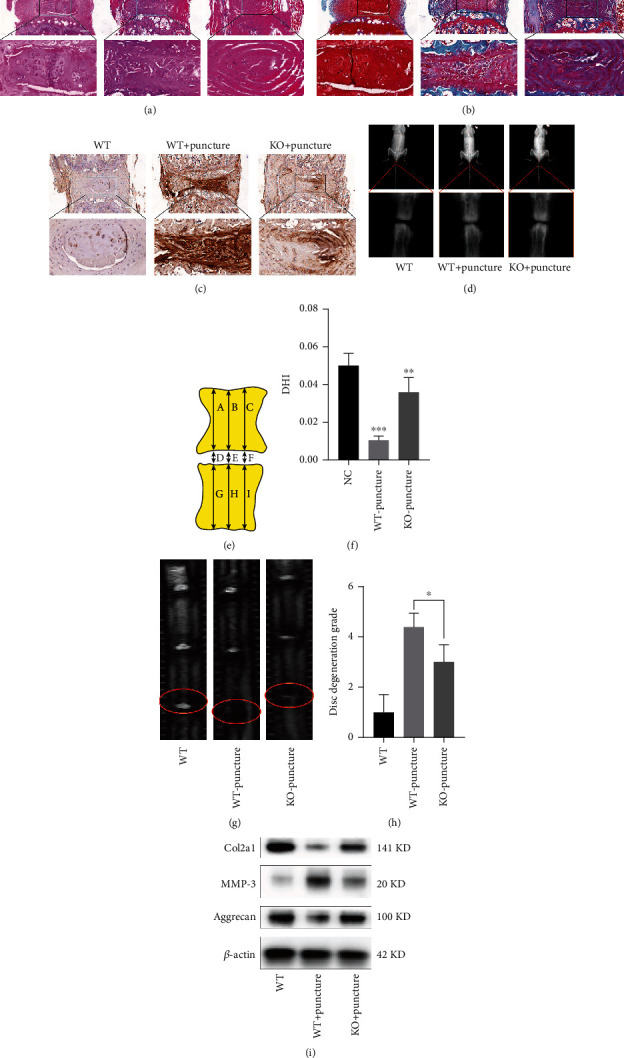
PREP knockout protects against puncture-induced IVDD. (a, b) HE and Safranine O/fast green staining showed the severity of IVDD changes at 6 weeks after puncture. Scale bar, 200 *μ*m. (c) Immunohistochemical staining of MMP-3 in mouse intervertebral disc samples. Scale bar, 200 *μ*m. (d) X-ray examination of mouse caudal vertebrae. (e) Schematic diagram of disc height index (DHI). DHI = (D + E + F)/(A + B + C + G + H + I). (f) The DHI of mice in different groups. Results are shown as mean ± SD, *n* = 5, ^∗∗^*P* < 0.01, ^∗∗∗^*P* < 0.001.

To further study the role of PREP in vivo, we produced PREP knockout mice. We established a mouse model of IVDD by puncturing the intervertebral discs. Compared to the WT group, the PREP-KO group had better histological morphology six weeks after the establishment of the IVDD model, as proved by Safranine O/fast green staining and HE staining (Figures 6(a) and 6(b)). Immunohistochemical results showed that the intensity of MMP3 in the intervertebral disc tissue of mice in the PREP-KO group was reduced compared to that in the WT group (Figure 6(c)). An X-ray examination was performed to detect the height of the punctured intervertebral disc (Figure 6(d)). The disc height index (DHI) was significantly increased in the PREP-KO group compared to that in the WT group (Figures 6(e) and 6(f)). Furthermore, MRI examination indicated that the Pfirrmann score of the PREP-KO group was significantly lower than that of the WT group, indicating less severe IVDD (Figures 6(g) and 6(h)). Western blotting showed that PREP knockout in mice promoted the expression of Col2a1 and aggrecan and decreased the expression of MMP-3 (Figure 6(i)).

## 4. Discussion

LBP has become a common disease that affects the physical and mental health of elderly people, causing a great economic burden on society [27, 28]. IVDD is a primary cause of LBP [29]. Although the molecular mechanism of IVDD remains unknown, the loss of collagen and water in NP is related to degeneration. Elaborating on the molecular mechanisms of apoptosis in NP cells will help develop new preventive and therapeutic measures for IVDD.

PREP has been studied as an important therapeutic target in many diseases. Becker et al. showed that Alzheimer-associated cerebrospinal fluid fragments of neurogranin are associated with PREP [30]. D'Agostino et al. proved that PREP deficiency impairs spatial learning and memory [31]. Kim et al. reported that hypothalamic PREP regulates pancreatic insulin and glucagon secretion in mice [32]. Moreover, PREP has been associated with many physiological processes. Myöhänen et al. reported PREP-induced angiogenesis [33]. Kilpeläinen et al. showed that PREP is related to autophagy [34]. As a cytoplasmic enzyme, PREP combined with MMPs can convert collagen into proline–glycine–proline fragments [21]. Therefore, we investigated the association between IVDD and PREP and found that the expression of PREP was significantly increased in degenerated disc tissues. The results were also confirmed by bioinformatic analysis of GSE146904 and GSE167931.

The bioinformatics analysis also suggested that the PI3K/AKT signaling pathway was altered between normal disc tissues and degenerated disc tissues. The PI3K/AKT signaling pathway is closely related to IVDD [35–37]. Activation of the PI3K/AKT signaling pathway in the NP further protects NP cells from apoptosis [38]. Wang et al. reported that the activation of the PI3K/AKT signaling pathway extended the degeneration of NP [37].

ROS act as critical mediators in regulating the ECM, autophagy, and apoptosis in NP cells [10]. The antioxidant capacity of the intervertebral disc is reduced as the antioxidant proteins in the degenerated intervertebral disc tissue decrease, leading to an imbalance of redox in the disc cells [16].

Etelainen reported that PREP inhibition could reduce oxidative stress by reducing the activity of NADPH oxidase; this indicates a connection between PREP and oxidative stress [39]. Next, we investigated the changes in the PREP and PI3K/AKT signaling pathways under TBHP stimulation. Stimulation with low concentrations of TBHP activated the PI3K/AKT signaling pathway and downregulated the expression of PREP. To determine whether PREP is directly related to the PI3K/AKT signaling pathway, we added the PI3K inhibitor LY294002 or agonist 740 Y-P to the NP cell culture medium. The expression of PREP in NP cells was significantly increased under LY294002 stimulation but suppressed under 740 Y-P stimulation. PCR results indicated that blocking of the PI3K/AKT signaling pathway with LY294002 restored PREP to a higher level under TBHP stimulation. This suggested that stimulation with TBHP may downregulate the expression of PREP through activation of the PI3K/AKT signaling pathway.

It has been proved that ROS mediated the expression level of AKT. Under moderate increase in ROS levels, AKT protein is activated. When ROS levels are high, AKT protein is inactivated [40]. Mistry et al. had reported that 10 *μ*M H_2_O_2_ promoted the expression of p-AKT [41]. Dimozi et al. showed that p-AKT protein was decreased with the treatment of 500 *μ*M H_2_O_2_ for 6 hours [42]. In our study, the PI3K/AKT pathway is activated to counteract moderate increased ROS levels. With the activation of PI3K/AKT pathway, the expression of PREP was reduced. However, when the intracellular ROS content exceeds its own clearance limit, oxidative stress occurs, and the PI3K/AKT pathway is inhibited which causing the increased expression of PREP. Since the PI3K/AKT pathway responded differently to low and high concentrations of TBHP, PREP changes correspondingly.

To understand how the PI3K/AKT signaling pathway inhibits the expression of PREP, we reviewed the candidate protein downstream of AKT. P53 is considered a critical transcription factor that can also be suppressed by the PI3K/AKT signaling pathway [26]. The expression of p53 in intervertebral disc tissues was positively correlated with that of PREP. Moreover, the overexpression of P53 increased the expression of PREP, indicating that p53 may promote the transcription of PREP. Luciferase reporter experiments after the ChIP assay verified the binding and transcriptional activity of P53 in the PREP promoter regions.

In addition, we studied the effect of PREP on apoptosis in NP cells. Knockdown of PREP attenuated high concentrations of TBHP-promoted apoptosis in NP cells, whereas overexpression of PREP increased the rate of apoptosis. Moreover, ECM secretion by NP cells was inhibited by oxidative stress and restored after PREP silencing.

In vitro experiments confirmed that the overexpression of PREP promotes apoptosis in NP cells, whereas knockdown of PREP protects NP cells from apoptosis induced by oxidative stress. In addition, in vivo experiments demonstrated that the degree of puncture-induced IVDD in PREP-KO mice was greater than that in WT mice.

The present study has several limitations. PREP-KO mice are not conditional knockout mice, which may have unknown effects on the in vivo experimental results. Moreover, whether p53 is the most important gene between the PI3K/AKT signaling pathway and PREP remains unclear. Finally, the bioinformatics analysis may be limited by the version of the packages used in R studio.

## 5. Conclusion

The mechanisms underlying IVDD remain unknown and require further study. Our study confirmed that the increased expression of PREP during disc degeneration and changes in the expression of PREP in NP cells under oxidative stress may be related to the PI3K/AKT signaling pathway. We demonstrated that the silencing of PREP protects NP cells from oxidative-stress-induced apoptosis. The in vivo knockout of PREP attenuates puncture-induced disc degeneration, indicating that PREP may have significant value in the treatment of IVDD.

## Figures and Tables

**Table 1:** 

Gene	Primer	Sequence
PREP	F	GTTTTCCGAGAGGTGACCGT
	R	TGGATATGTTGAAGCCGCCA
Bax	F	TCATGGGCTGGACATTGGAC
	R	GCGTCCCAAAGTAGGAGAGG
Bcl-2	F	CTTTGAGTTCGGTGGGGTCA
	R	GGGCCGTACAGTTCCACAAA
GAPDH	F	AATGGGCAGCCGTTAGGAAA
	R	GCGCCCAATACGACCAAATC
P53	F	ACCTATGGAAACTACTTCCTGAAA
	R	CTGGCATTCTGGGAGCTTCA

## Data Availability

Data in this study would be available if required.
